# Peering into the Cuba phytosanitary black box: An institutional and policy analysis

**DOI:** 10.1371/journal.pone.0239808

**Published:** 2020-09-28

**Authors:** Demian F. Gomez, Damian C. Adams, Rosa E. Cossio, Paloma Carton de Grammont, William A. Messina, Frederick S. Royce, Sebastian Galindo-Gonzalez, Jiri Hulcr, Berta Lina Muiño, Luis L. Vázquez

**Affiliations:** 1 School of Forest Resources and Conservation, University of Florida, Gainesville, Florida, United States of America; 2 Purdue University, West Lafayette, Indiana, United States of America; 3 Water Institute, University of Florida, Gainesville, Florida, United States of America; 4 Florida Agricultural Market Research Center University of Florida, Gainesville, Florida, United States of America; 5 Agricultural and Biological Engineering Dept, University of Florida, Gainesville, Florida, United States of America; 6 Department of Agricultural Education and Communication, University of Florida, Gainesville, Florida, United States of America; 7 Entomology and Nematology Department, University of Florida, Gainesville, Florida, United States of America; 8 Instituto de Investigaciones de Sanidad Vegetal, Havana, Cuba; Canakkale Onsekiz Mart University, TURKEY

## Abstract

To mitigate the movement of non-native organisms with trade, phytosanitary systems have been implemented within and between countries. In some countries such as Cuba, little is known about the within-state plant health system. To facilitate the development of future trade partnership between Cuba and the United States, agencies need to understand the organizational structure and diagnostic capacity of the Cuban Plant Protection System, identify potential synergies between the United States and Cuban systems, and identify steps towards cooperation. This paper fills this critical void by presenting a descriptive analysis of the plant health system in Cuba. Information was integrated from available literature, informal interviews with Cuban experts, and workshops focused on Cuban policies, risk, and potential collaboration attended by Cuban and American experts. We identify the next practical steps in improving cooperation, including building trust and capacity. Mutual understanding of phytosanitary systems will be crucial for the regional economic and environmental stability of a post-embargo United States-Cuban relationship.

## Introduction

Invasive species are a threat to natural and working lands, including farms and forests, to the integrity of ecosystems in both rural and urban contexts, and to the sustainability of the economic systems that depend on ecosystem services. Losses from invasive species in the United States are estimated at more than US$120 billion annually [1,2]. Yet, these estimates are conservative because they do not include the environmental costs of added management, nor the increased crop losses that these exotic pests may cause [3].

Increasing international trade has led to an unprecedented movement and establishment of invasive species in recent decades [4], with one-sixth of the global landscapes classified as highly prone to invasions, including areas of developing economies and biodiversity hotspots [5]. In the United States alone, chestnut blight has eliminated chestnut trees [6], horticultural crops have been impacted by the light brown apple moth in western North America [7], dutch elm disease has killed elm trees across eastern North America [8], citrus greening and the brown citrus aphid have caused severe damage to the citrus industry [9,10], billions of ash trees are now dying due to the emerald ash borer across the East [11], and more than 300 million redbay trees have been killed by laurel wilt [12]. Other invaders are on the horizon but not yet arrived. For example, the Sweetgum Inscriber recently discovered in China is known to be lethal to American sweetgum trees [13].

International collaboration and coordination are critical to the management and prevention of invasive pests and diseases, a need that has been globally recognized. Today, 183 countries are contracting parties of the International Plant Protection Convention (IPPC), an international agreement between countries to control and prevent the spread of pests, where each country establishes a National Plant Protection Organization (NPPO) that determines and manages pest risk, and implements international guidelines on prevention and management [14]. While some countries have implemented efforts to comply with international phytosanitary obligations in order to secure trade, others are poorly prepared and lack the needed resources to enhance their plant protection systems [15].

The Caribbean Region is one of the most biologically diverse regions in the world. Because of its geographic proximity and ecological similarities to Florida, the region is a key pathway for potentially harmful exotic pests entering the United States. Despite its geographic proximity, Cuba, the largest Caribbean country and the closest large land mass to Florida, has been functionally isolated from the United States since the 1950s with the Cuban Revolution and the subsequent commercial, economic, and trade embargo. In 2000, the United States government passed the Trade Sanctions and Export Enhancement Act (TSRA) which, for the first time in nearly 40 years, allowed United States firms to sell agricultural and food products, and selected medical products to Cuba. Since the passage of TSRA, United States firms have shipped nearly $6 billion worth of food and agricultural products to Cuba and continue to sell hundreds of millions of dollars worth of goods to Cuba each year [16].

The Cuba-Florida pathway has been recognized as a significant potential gateway for pest exchange between Cuba and the United States, being described as a blind spot in the United States biosecurity continuum [17]. Very little is known in the United States about Cuba’s institutional stance and capacity regarding invasive species threats. Effectively, the largest country in the Caribbean remains a ‘black box’ to state and federal phytosanitary authorities in the United States. This situation is untenable given the risk of pest and disease transmission to or from Cuba through trade, tourism, and hurricanes and other pathways. The risks posed are likely to increase over time with the climate change-mediated rise in extreme weather events, and the potential reestablishment of open trade [18]. Still, responses need to be proportionate to the risks. For example, it has been estimated that an hypothetical introduction of a wood boring beetle from Cuba could cause an economic damage to the United States of US$ 2.4 million [18].

National capacity will ultimately determine regional and global capacity for plant resources protection [19]. Cuba represents a potential pathway for new pest and disease introductions in the Caribbean region, including islands and the surrounding coasts. The ‘black box’ status of Cuba in the Caribbean thus poses a critical threat to plant health, not just to the United States but globally. For instance, in 2003 a study on capacities to face invasive species threats in the Caribbean was conducted, but information from Cuba could not be gathered [20]. In recent decades, local efforts have been conducted to document the Cuban system [21–23], but those publications are not easily accessible outside Cuba.

Efforts to strengthen plant health systems through collaboration, including regulatory frameworks, are underway in many regions. Two initiatives exist in the Caribbean and Central America: 1) the Greater Caribbean Safeguarding Initiative (between the United States Department of Agriculture’s Animal and Plant Health Inspection Service-APHIS and some countries from the Caribbean Region); and 2) International Regional Organization for Plant Protection and Animal Health-OIRSA (Regional Plant Protection Organization for Central America and Dominican Republic). These initiatives aim to protect their territories and economies from the introduction and spread of high-risk pests through collaborative efforts, regulatory commitments, and information exchange. However, Cuba is not part of these initiatives, missing potential benefits from the different collaborative agreements.

Very recently, an increase in dialogue between Cuban and United States researchers created an opportunity to build mutual understanding of phytosanitary institutions and policies. In 2015, a joint statement on cooperation for environmental protection was signed between the United States and Cuban governments to increase cooperation and facilitate the exchange of scientific information [24]. Notably, in 2016, the USDA and the MINAG (Cuban Ministry of Agriculture) signed a Memorandum of Understanding (MoU) with the objective of establishing bilateral cooperation in the agricultural and forestry sectors, including plant health issues, standards of agricultural trade, and scientific exchange [25].

With the Obama administration’s resumption of diplomatic relations with Cuba, United States regulations began to evolve in ways that provided opportunities for export of a small range of goods from Cuba to the United States. Currently, only charcoal is allowed to be exported from Cuba to the United States. Despite the fact that we expect trade to increase over time, we lack the data needed to predict the arrival of new plant pests from Cuba or to plan a response. This also poses a major dilemma for Cuba—even if the United States economic embargo were lifted immediately, it may take a decade or more to build the regulatory apparatus significantly expand certain commercial activities with Cuba, including sales of fresh fruits and vegetables. In order to harmonize both systems and reduce potential delays, United States agencies need to: 1) understand the organizational structure and analytic capacity of the Cuban Plant Protection System; 2) identify potential synergies between the United States and Cuban systems; and 3) suggest future steps for cooperation.

This paper fills this void by presenting a novel descriptive analysis of the plant health system in Cuba. This review integrates information from literature, informal interviews with Cuban experts, and workshops focused on Cuban policies, risk, and potential collaboration attended by Cuban and American experts. The historical context of the phytosanitary system in the country is provided, followed by the description of its organizational structure. Secondly, Cuba’s integrated pest management (IPM) programs, pest surveillance, and strengths and shortcomings of the system, are described. Finally, synergies between the Cuban and United States plant protection systems are identified to define next steps in a future collaboration, focusing on sharing information, identifying potential threats, and developing effective rapid response strategies. This paper contains the first published, detailed description and analysis of the Cuban plant health system.

## Materials and methods

IRB protocol 201601213 approved by the University of Florida Institutional Review Board under the project "The Emerging Cuba-US Plant Pest Pathway". Data were collected and analyzed using qualitative methods that included analysis of both primary and secondary sources of information following an exhaustive search of the scientific and gray literature in the United States and Cuba. Literature included reports, government documents, and other creditable sources of information. Data collection protocols were employed pursuant to the Cooperative Agreement between the University of Florida and the Cuban Plant Health Research Institute (Instituto Nacional de Investigaciones de Sanidad Vegetal—INISAV). The Strengths, Weaknesses, Opportunities and Threats (SWOT) analysis framework was applied to categorize significant factors of phytosanitary institutes with experts’ opinions as a precursor to strategic planning, decision making, and action [26]. The SWOT framework was composed of internal and external assessments. The internal assessment was conducted to identify strengths and weaknesses of the phytosanitary system, whereas the external assessment was applied to discover opportunities for future collaboration [27].

Literature reviewed for this study, made possible via the cooperative agreement, included publications from Cuban journals, reports and whitepapers, booklets, flyers, law decrees and legal resolutions. Many such sources were only available in hard copy and in Spanish. Observational data were collected via in-person meetings in Cuba between Cuban and United States scientists and agency staff (October 2016, April 2017 and September 2018). Information was gathered through unstructured discussions with a wide range of Cuban plant health scientists and experts on the basis of their knowledge and experience on the topic [28]. These meetings provided unique opportunities to observe: 1) agricultural settings (fruticulture cooperatives from Artemisa and Matanzas Provinces managing more than 12,000 ha of avocado, citrus, mango, and guava) and management operations presented by field technicians during the VIII International Scientific Seminar of Plant Health (April 2017); and 2) Cuban pest identification capacity using invasive wood borers as an indicator, by visiting reference collections, workshops, and trainings on identification and collecting wood borers from 2016 to 2018.

Finally, two facilitated expert workshops provided opportunities to assess experts’ attitudes and perceptions of main strengths and shortcomings of Cuban (and United States) policies and institutional arrangements to prevent the movement of plant pests. One was held in the United States at the University of Florida and one in Cuba at INISAV. These workshops allowed to corroborate existing data and to generate new data on perceptions on areas of potential synergies and gaps. We used the SWOT framework during the workshops to categorize significant factors of the phytosanitary institutes as a precursor to strategic planning, decision making, and action [26,29]. The workshop at the University of Florida was attended by 14 experts, including key representatives from the Animal and Plant Health Inspection Service (APHIS) of the United States Department of Agriculture (USDA) and the Division of Plant Industry (DPI) of the Florida Department of Agriculture and Consumer Services (FDACS), as well as scientists from the University of Florida. The workshop at INISAV was attended by 18 experts, including scientists from INISAV as well as technicians in charge of implementing the phytosanitary policies at the provincial level in Cuba (Laboratorios Provinciales de Sanidad Vegetal).

## Results

### Evolution of cuban plant health system and historical context

The plant health system in Cuba has evolved under the influence of deep changes in the country’s agrarian policies and economic situation, as well as international trends. Agricultural land has experienced noticeable changes, evolving from a monoculture and export-oriented system (based on sugarcane and intensive agriculture) toward inclusion of the agro-ecological sector based on low industrial/imported input usage (based on crop diversification and chemical pesticide reduction). Vázquez [23] classifies the evolution of the system in four main periods: 1) high yield and agrochemical period until 1974; 2) the crisis of the conventional agriculture between 1975 and 1985; 3) the later implementation of IPM protocols; and 4) and the agro-ecological paradigm after 1992 with focus on diversification of agriculture.

The creation of the Agronomic Central Station in Santiago de las Vegas during 1904, set the foundations for a plant health service in Cuba, establishing the first official plant health service with Sanitary Police and Phytopathological Supervision Services in 1913 [21]. In 1914, the appearance of the citrus blackfly (*Aleurocanthus woglumi* Ashby) and the sugarcane mosaic virus (Potyviridae) on Cuban agricultural exports, resulted in firm regulations from the phytosanitary authorities of the United States Department of Agriculture (USDA). As a response, a Phytopathology Commission was created in Cuba, and two years later, the Plant Health Commission emerged to monitor the Phytopathology Commission’s work and to establish control measures for the citrus blackfly. In 1919, the Plant Health Commission was restructured as the Plant Health Office (Oficina de Sanidad Vegetal) to issue export certificates for plants and fruits, to prevent blackfly spread, and to assemble an inspection service in post offices, ports and railways [30]. The effectiveness in pest control achieved by the Plant Health Special Office led in 1931 to the implementation of new legislation that governed all phytosanitary protection activity. During the 1940s, the Quarantine Advisory Committee was created to grant import permits and address quarantine issues, increasing border control personnel across the country in ports of entry and warehouses by the 1950s [21].

During the first half of the 20^th^ century, the agrarian system in Cuba was dominated by large scale sugar production, driven in large measure by foreign capital [31], with 73% of the land owned by less of 10% of the landholders by 1959 [30]. After the Cuban Revolution in 1959, two Agrarian Reform Laws transformed the agrarian system, abolishing large sugar and cattle landholdings, granting land access, technical support, allowing low interest credits to agricultural workers, and creating Agricultural Cooperatives [32–34]. In the 1970’s under the ‘influence of the green revolution’, large specialized and highly mechanized companies were created, utilizing high volume of agrochemicals, with high yields as the main objective [23]. The phytosanitary management board of the National Institute of Agrarian Reform (INRA) installed a Central Phytosanitary Laboratory, currently known as the Plant Quarantine Central Laboratory (Laboratorio Central de Cuarentena Vegetal—LCCV), responsible for examining all imported vegetables and farm samples and diagnosing problems. During the 1960s, chemical applications of pesticides were based on fixed calendars; thus, by the end of the decade, intensive agriculture and synthetic pesticides usage had caused severe social, economic, and environmental impacts [22]. From 1975 to 1985, concerns about the ineffectiveness of chemical control alone and the emergence of new pests contributed to strengthening the plant health system with the development of protocols for pest monitoring, and targeted chemical control. By the mid 1970s the Cuban State Plant Protection System (SEPP) was established [35], composed of the Ministry of Agriculture (MINAG)’s National Center for Plant Health (CNSV), the agrarian productive sector (including state farms and cooperatives), and scientific and educational centers.

In 1982, the Cuban government implemented IPM as its official policy, and a strong effort ensued to replace synthetic insecticides with biocontrols by the mid 1980s [30,35]. The main challenge in this process was to familiarize phytosanitary technicians and farmers, used to programmed and indiscriminate broadcast applications of chemical broad-spectrum pesticides, with the conceptual changes needed for the proper implementation of an IPM program [36]. From 1985 to 1992, phytosanitary policy focused on the development of IPM programs, through the consolidation of the ‘pest signaling methods’ for monitoring and implementation of the National Program for Production of Biological Control. Scientists, technicians, and producers began to search for alternatives to the high input agriculture practiced in the country [37,38]. In 1988, the National Program for Production of Biological Control established a network of laboratories called Centers for the Reproduction of Entomophages and Entomopathogens (Centros Reproductores de Entomófagos y Entomopatógenos—CREEs) [39]. In 1991, the MINAG and the Ministry of Sugar reviewed this National Program creating 176 CREEs, biopesticide plants, and a pilot plant for development of new technologies [30,38]. During this decade, the Cuban agricultural sector, mainly State owned, had dedicated cultivable land to three export monocultures (sugarcane, tobacco, and coffee) and was highly dependent on external resources [40].

During the 1990s, bioinsecticides and insect natural enemies were mass produced, and natural pesticides based on plant extracts were developed [41]. With the collapse of the socialist block and the loss of Soviet preferential trading arrangements and subsidies, the Cuban government declared a ‘Special Period in Time of Peace’ (*Período Especial*), signaling an austerity program, setting up a national strategy to reorient its international trade [42], and transforming the agricultural sector to a low-input, self-reliant and diversified system, that encouraged the development of local agro-ecological technologies [33,34]. The collapse of the Soviet Union together with the United States trade embargo produced a shortage of synthetic fertilizers and pesticides, transforming urban agriculture into national priority with focus on sustainability [43].

Nineteen ninety-two signaled the start of the ‘agro-ecological paradigm’ period, distinguished by agriculture diversification, and widespread adoption of agro-ecological pest management (APM) with reduction in synthetic pesticide use and significant changes to phytosanitary management practices [23]. This approach to agriculture in Cuba was shaped by the need to produce high yields with minimal use of external inputs, especially agrochemicals derived from fossil fuel [43]. Biopesticides and natural enemies became the basis of pest control, chemical fertilizers were often substituted by biofertilizers, animal traction was expanded, and soil restoration and conservation was prioritized [22,37]. These practices facilitated a reduction in the use of synthetic pesticide of 50% after the first year [22]. Furthermore, a strong movement of urban agriculture arose, with thousands of families producing food through organic methods and in organic urban cooperative gardens known as *organopónicos* [38], proved to be ideal for growing crops on poor soils.

The ‘agro-ecological paradigm’ period was also marked by changes in land tenure and management, strengthening of the cooperative system, and the emergence of urban and sub-urban agriculture that emphasized local expertise. Agro-ecological pest management was strongly integrated into the MINAG’s Urban, Suburban and Family Agriculture Program, and prioritized by this Ministry [44]. In 2011, Cuba announced the adoption of the Lineamientos de la Política Económica y Social del Partido y la Revolución (Guidelines of the Economic and Social Policy of the Party and the Revolution), new economic and social guidelines to continue the improvement of economic and social system, which included policies intended to strengthen the agricultural sector by reducing unproductive lands, increasing yields through crop diversification, rotation and polyculture, including phytosanitary protection, and enhancing production and use of organic fertilizers, biofertilizers and biopesticides [45]. Those changes involved research and development of new pest management methodologies, as well as the implementation of policies and regulations to address changes in its plant health system and international agreements, including the adhesion to the IPPC [46].

### The Cuban State Plant Protection System

#### Organizational structure

**Fig 1:**
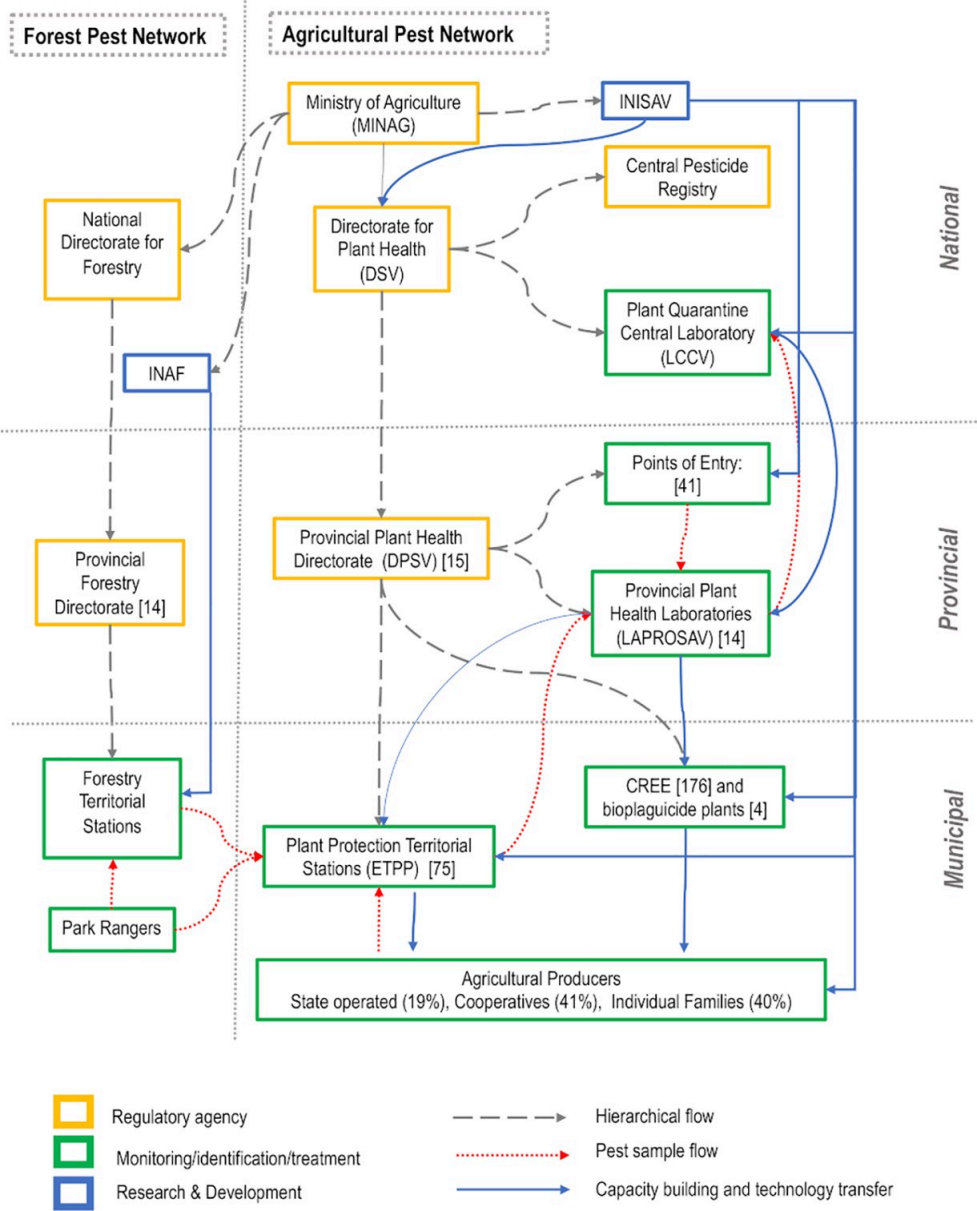
Model of the Cuban State Plant Protection System (SEPP). Regulatory agencies, research institutes, and surveillance institutions are highlighted. Early detection systems and pest identification flow are shown for agricultural and forest systems. Arrows show hierarchical flow, pest samples movement, and capacity building flow.

The current Cuban State Plant Protection System (Sistema Estatal de Protección de Plantas, SEPP) organizational structure is characterized by its multi-institutional, multisectoral, and decentralized nature (Fig 1). This structure allows the system to have a strong and dynamic presence across the country, establishing close collaboration with the agricultural producers, who actively cooperate and support early detection, survey, management and eradication of pests.

The plant health system is regulated by the Ministry of Agriculture (MINAG) through its Directorate for Plant Health (Dirección de Sanidad Vegetal—DSV, previously Centro Nacional de Sanidad Vegetal—CNSV). The DSV serves as the country’s NPPO accountable to ensure phytosanitary protection through plant quarantine, plant protection (phytosanitary surveillance, phytosanitary emergencies, and response plans), technical development, and registration of pesticides. The DSV regulates and overseas the Plant Quarantine Central Laboratory (Laboratorio Central de Cuarentena Vegetal, LCCV*)*, the Central Pesticide Registry, and 14 Provincial Plant Health Directorates (Direcciones Provinciales de Sanidad Vegetal, DPSV). The LCCV provides scientific support to the plant system, conducting final diagnosis of regulated quarantine species and research. The Central Pesticide Registry regulates the testing, commercialization, and use of new products across the country, focusing on social and environmental responsibility.

The DPSVs are in charge of regulating plant pests and diseases within the limits of their province. They are equipped with five administrative units: 1) Plant protection, 2) Quarantine, 3) Biological Control, 4) Training and 5) Biostatistics and Pest surveillance. Each DPSV also supervises and provides technical support and training to the following institutions within their province: a Plant Health Provincial Laboratory (Laboratorio Provincial de Sanidad Vegetal—LAPROSAV), Points of Entry (Puntos de Entrada- PI), Plant Protection Territorial Stations (Estaciones Territoriales de Protección de Plantas—ETPPs), Centers for the Reproduction of Entomophages and Entomopathogens (Centros Reproductores de Entomófagos y Entomopatógenos—CREEs), and biopesticide plants.

The LAPROSAVs and the ETPPs represent two key institutions within the plant health system, particularly because they are the institutions in charge of pest monitoring, diagnosis and management, working in direct collaboration with farmers and providing the first line of control. The LAPROSAVs, present in nearly every province of the country, and the ETPPs, distributed across the territory as the SEPP base-level units, are responsible for pest diagnosis and surveillance, capacity building, transference of technology at the local level, development of novel pest management practices, and recommendations on pest control measures. Moreover, the ETPPs are responsible for conducting phytosanitary inspections and surveys (75% of commercial crops), and the implementation of the ‘signaling and forecasting system’, a surveillance-based decision-making process that comprises the basis of IPM and APM programs [47].

Located across the country, CREEs have increased the local production of biopesticides and biological control agents, selling products to the entire agronomic sector, including small farmers. Together with the biopesticide plants, more than 120 tons of biopesticides and biological control agents per year are produced and distributed across the country, reducing significantly the need to import synthetic pesticides [48].

The SEPP articulates with a wide range of research institutes under the MINAG, including the Sugarcane Research Institute (Instituto de Investigaciones de la Caña de Azúcar—INCA), Soils Institute (Instituto de Suelos—IS), the Agro-Forestry Research Institute (Instituto de Investigaciones Agro-Forestales—INAF), the Tropical Fruticulture Research Institute (Instituto de Investigaciones de Fruticultura Tropical—IIFT), the Tobacco Research Institute (Instituto de Investigaciones del Tabaco—(IIT), and the INISAV among others [49]. The Agro-Forestry Research Institute, is responsible for all scientific, and technical aspects of the forestry industry and forest products, improving silviculture, forest protection, capacity building, and developing new technologies for the diversification of forest products [50]. Forest protection in Cuba is under the responsibility of MINAG, with direct technical assistance from the Forestry Experimental Stations. Seven Forestry Experimental Stations distributed across the country keep pest inventories and conduct local research, closely interacting with ETPPs when phytosanitary problems arise [51].

To provide scientific and technical support to the SEPP, the INISAV was created in December 1976 and has since contributed significantly to the coordination of the national scientific community. Through a network of laboratories, INISAV research targets the country’s agricultural phytosanitary problems, including the study of quarantine pests and massive rearing of biocontrol agents, used by CREEs and biopesticides plants [52]. Moreover, INISAV addresses quality control of all chemical pesticides registered in Cuba through its Quality Control Laboratory, that works in coordination with the LAPROSAVs and provides technical advice to the Central Pesticide Registry [53]. Residual analysis of organophosphates, carbamates, dithiocarbamates, triazoles, and pyrethroids, are conducted for various export products (e.g., honey, citrus, coffee) [54].

The Ministry of Education, accountable for primary and technical education, has a network of 74 agrarian polytechnic institutes, with agrarian production facilities for students’ practical training and capacity building on plant health issues [38]. The Ministry of Higher Education (MES), responsible for university and postgraduate education, oversees all agrarian universities, plus some important research institutes and experimental stations. In this way, the MES helps to provide the scientific, technical, and methodological basis for pest management in the country, and has significant influence on agricultural policy. The plant health service actively establishes collaborations with agricultural universities to attract young students for thesis and practical work at ETPPs and LAPROSAVs, with the possibility of a job opportunity after graduation. Major Cuban universities offer plant health majors and experienced professors from these centers collaborate with the SEPP on research projects and training activities. Universities offer opportunities for capacity building to the SEPP’s specialists so that they can pursue graduate degrees. Cuba has 11 percent of the scientists in the Caribbean region, with more than 140,000 high-level professionals and medium-level technicians [55].

### Pest management programs in Cuba

Cuba has more than 25 pest management programs, with two different approaches [44]. For intensive crops (e.g., potatoes, tomatoes), an IPM scheme is used, integrating synthetic pesticides with biological control. The other approach focuses on crops grown on small farms and urban agriculture and uses agro-ecological management (APM), including biological control, biopesticides, agronomic practices (e.g., crop rotation, polycropping, soil covers), and functional integration of auxiliary vegetation structures.

The efficiency of the SEPP is based on its multi-institutional collaboration and the scientific support of the INISAV, allowing a dynamic presence across the territory. As a complement to the technical infrastructure of the SEPP, a governing document ‘Guidelines to SEPP’s Functions and Procedures’ was elaborated, addressing all phytosanitary activities across the territory [56]. While INISAV develops pest management methodologies, the LAPROSAVs validate the procedures, and the ETPPs implement them in close collaboration with agricultural producers. Subsidized scientific-technical phytosanitary services are provided to farmers, including phytosanitary diagnosis, signaling and forecasting, pest management, and biological control [22]. ETPPs technicians provide direct technical assistance to farmers during the adoption of prevention and control practices, with focus on agro-ecological methods and minimum use of synthetic pesticides. These methods, integrated into Pest Management Territorial Programs, are specific for different agricultural systems and crops of economic importance [22,23].

#### Pest diagnosis and early detection

Pest diagnostics and early detection involves identification of native and exotic plant pests and their natural enemies, including phytosanitary analysis of seeds, seedlings, and soil. Samples that require identification are received at the ETPPs, usually brought by agricultural producers, whether State operated, cooperatives, or small farmers, following specific sample collection instructions provided by the ETPPs’ specialists. If needed, ETTP specialists can inspect the farm to confirm a diagnosis and provide recommendations. For forest pest diagnosis, Forest Rangers conduct surveillance, notifying the ETPPs and the Forestry Territorial Stations when a phytosanitary problem is detected, which will conduct the sampling and further identification. If the organism cannot be identified at the ETPP, the sample is sent to the LAPROSAV. Phytosanitary specialists, located on each LAPROSAV, are trained and updated periodically, with availability of collections, taxonomic keys, and monographs [22]. When samples cannot be identified at the LAPROSAVs, they are sent to the LCCV for final identification.

Quarantine phytosanitary inspectors, located at the 41 Points of Entry (i.e., ports, airports, and their surrounding areas designated in the 1980s), conduct pest diagnosis in their respective provincial laboratories for quarantine pests (from official list of regulated pests) following standardized international procedures. Reference material, such as taxonomic keys and entomological collections, support pest diagnosis [21]. Final diagnosis is performed typically in the LCCV, but can also be conducted by the LAPROSAVs or inspectors at Points of Entry for common species (e.g., storage pests). However, while preliminary diagnosis can be conducted by the LAPROSAVs and at Points of Entry, quarantine pests are always sent to the LCCV for final identification.

#### Pest surveillance

Pest surveillance, under the responsibility of the ETPPs, is done through what the Cubans call ‘Signaling and Forecasting System’ (Sistema de Señalización), considered the basis of Cuba’s agroecological approach to reduce chemical pesticides. Focusing on population dynamics, economic thresholds, weather, and natural enemies, pests are periodically monitored through a designated sampling method that includes surveillance of fixed plots and transects for each economically important crop. The economic thresholds, also referred as action thresholds, have been developed locally for different crops [57]. Pest records, together with phenological and meteorological data are analyzed locally at each LAPROSAV to model these economic thresholds, and when appropriate, a warning is issued to farmers about the pest problems to address, ensuring a rapid response when needed [56,58]. In this process, information on relevant pests, monitoring systems, and population thresholds to start mitigation measures, are provided to farmers. Depending on the crop, different mandatory management recommendations are given to farmers, providing technical support. However, farmers are not always properly served by specialists, with lack of specific training on applications and knowledge on the use of protective equipment [59]. Supported by quarantine defense programs and the survey system which processes approximately 35,000 samples per year, the SEPP guarantees the inspection and surveillance in 75% of agricultural areas, all quarantine stations, and urban areas [47]. In forest systems, the signaling and forecasting is based on the ETPP specialists working together with forest managers and staff to carry out sampling, phytosanitary inspections, and pest diagnosis.

#### Plant quarantine system

The plant quarantine system is divided into an Interior Quarantine and an Exterior Quarantine, with a responsible chairman for each Department in each Province. Exterior quarantine is related to Points of Entry, including import inspections, export certificates (to authorize shipments), and surveillance in surrounding areas to ports and airports. Since 1996, External Quarantine work has been extended to cover also free-trade zones and industrial parks [21]. Phytosanitary import permits, including an international phytosanitary certificate issued by the official authority in the country of origin (according to IPPC), are required for imports of plant products, where the DSV conducts a pest risk analysis and issues the import permit when approved. All authorized imports are overseen by the DSV inspectors who will corroborate the fulfillment of import requirements in the country of origin, cancelling imports in cases of non-compliance [47]. The SEPP guarantees the inspection of 100% of agricultural loads that arrive in Cuba, as well as warehouses and seed production areas [21,47]. As a contracting party of the IPPC, Cuba recognizes three groups of pests: quarantine pests A1 (pests not present in Cuba), quarantine pests A2 (pests that are present in Cuba but regulated under official control), and A3 regulated non-quarantine pests (non-quarantine pests which produce an economic impact and therefore are regulated for compliance with the importer country). The updating of pest lists is done as soon as the need for modifications is identified to assist contracting parties in issuing the correct phytosanitary certificates.

Interior quarantine, on the other hand, is responsible for surveillance of quarantine pests, addressed at the municipal level through the ETPPs’ technicians. This involves inspections of nurseries, seed orchards, research stations with focus on exotic crops, warehouses of processed products, and export areas. Moreover, technicians conduct follow-ups on potential threats recognized by quarantine inspectors at Points of Entry, and emit phytosanitary export certificates.

#### Biological control

The existence of a biological control program is possible because of the network of CREEs and biopesticide plants, prioritized in the Cuban plant health system. Natural enemies and insect pathogens are massively reared and released across the territory, based on agricultural production needs in each region. Quality control is ensured by national regulations, controlled by the LAPROSAVs at the field level, and supplying certified strains [35]. Each LAPROSAV has specialists in entomophages and entomopathogens, as well as a chief of the biological control program, responsible for supervision of methodologies and quality control of products at the CREEs [58]. Moreover, the ETPPs advise farmers on biological-control use and effectiveness monitoring. However, INISAV, in cooperation with LAPROSAVs and ETPPs, is responsible for the development of all the certified strains and new technologies to implement.

#### Chemical control

Chemical control strategies, widely used during the 1970s, have been minimized after the development of monitoring systems, biological control strategies, and the agroecological approach. Nevertheless, there is a widespread desire to use more agrochemicals among farmers, especially synthetic fertilizers, contradicting the perception of Cuba’s focus on agroecology [60]. Synthetic pesticides are still used regularly for intensive crops, such as potato and tomato [23]. Moreover, and despite ecological awareness of their risks, farmers occasionally use synthetic pesticides in urban settings because of high efficiency and little work effort.

Despite Cuba’s advances in agroecology have been highlighted in the last decades, reliable statistical data on agrochemical use is scarce [61]. The use of agrochemicals is restricted and availability of agrochemicals on the free market is limited [43]. Moreover, the absence of adequate synthetic pesticides in Cuba has produced great economic losses due, for example, to thrips, severely impacting crops of beans, potatoes, and peppers [62]. Biological control is not enough to control this pest, and synthetic pesticides are an important component of the IPM strategy [63].

#### Capacity building

The Cuban plant health’s capacity, as well as the capacity building program is considered one of the strongest sources of support to agriculture in the country [22]. The SEPP is staffed by technicians and professionals graduated from different Cuban agricultural polytechnic institutes, research institutes and universities, with constant training and technical updates to improve the system’s capabilities. During the 1970s and 80s, scientists were trained through extensive exchange programs with the Soviet Block. Currently, annual seminars and courses are conducted for farmers, as well as for LAPROSAVs and ETPPs. Moreover, a series of professional events are offered from one to four years, such as national scientific-technical events for agricultural professionals and researchers, technological forums, international scientific seminars on plant health, and international symposia on phytosanitary surveillance. Training campaigns on prevention of invasive organisms have been relevant for phytosanitary programs. Every one or two years, a national entomology course is organized between INISAV and the Plant Quarantine Department to promote scientific-technical exchange. Moreover, the agricultural producer population is highly educated in comparison with other lesser-developed countries. Literacy level of farmers has been essential for a proper implementation of the system, as knowledge and information on methodologies reaches all the agricultural sector, supported by exchange programs facilitated by the farmer cooperatives.

Exterior Quarantine phytosanitary inspectors, with an agronomy background, receive special training before starting their activities at their base units. New inspectors will typically receive plant health and professional development courses, taught every one or two years at the national level with focus on methodologies, procedures, legislation, and quarantine pests. Moreover, LAPROSAVs’ specialists (i.e., entomologists, nematologists, virologists, pathologists) will train External Quarantine inspectors on pest identification, with focus on quarantine pests. Newly hired LAPROSAV entomologists are trained in different LAPROSAVs and in the LCCV, where they receive training on pest identification and procedures. ETPP specialists have a similar training program to External Quarantine phytosanitary inspectors, but with a focus on crop pests. Phytosanitary technicians from state companies and cooperatives, usually farmers or workers with advanced scientific and technical knowledge, are periodically trained by ETPP specialists on plant health issues. Moreover, they play an important role in phytosanitary surveillance and implementation of technologies at the local level, with an average of five to seven technicians per 645 km^2^ [58].

### Shortcomings of the Cuban plant health system

Despite the strong organizational structure, some aspects of the Cuban phytosanitary system constrain its full potential. Key problems include laboratories with obsolete equipment, migration of young professionals to other job opportunities, and lack of digitized data for the SEPP. Only a few laboratories are stocked with up-to-date equipment (e.g., the Plant Quarantine Central Laboratory and the INISAV’s Chemistry Scientific and Technical Base Unit lab), whereas most laboratories have either obsolete equipment (from the 1980s), or a complete lack of equipment. Financial constraints limit the purchase of modern equipment in most laboratories. Nevertheless, in institutions like INISAV for example, personnel from the Department of Quality, Normalization and Metrology conduct annual certifications to the entire institute’s equipment to ensure proper operation. In recent years, young professionals from the plant health system, in particular from INISAV, have been leaving the institute to pursue better paid positions in the private sector. Therefore, most professionals with scientific and technical expertise are quite senior or retiring.

One important issue is that SEPP’s data has not been completely digitized yet. In 2009, the Center for Plant and Animal Health in collaboration with the DSV developed the software FITOVIGIA to support phytosanitary surveillance in the country [64]. However, due to technological differences amongst ETPPs, the use of this software was not implemented and was withdrawn. During 2017, INISAV started the implementation of the National System for Management of Phytosanitary Information (INFOSAV), in order to integrate information and communication technologies to capture, store and analyze plant health data, as well as multiple environmental variables (climatology, edaphology, and hydrology) using GIS tools. Currently, national plant health data is still being digitized into Excel datasets, with plans to implement a database system in the near future. However, the lack of internet access, combined with the lack of technology, slows down the process.

It is unclear whether pest management programs meet their goals. For example, biocontrol programs are widely utilized and publicized. Since its implementation, more than 200,000 hectares are treated every year, with 60% reduction in imported synthetic pesticides [65]. However, despite a national environmental strategy that stated that 80% of the pests and diseases control should be conducted with biopesticides by 2010 [59], no reports on compliance, nor reports on efficacy, exist or were provided to external evaluators. Moreover, agrochemicals are used at unjustified times with inadequate doses, resulting in failed pest control. Despite the technical support provided on agricultural management, farmers are not properly trained on chemical control applications, causing health risks for workers and nearby residents, together with environmental impacts. A recent study conducted in Sancti Spíritus, a province focused in agricultural production, showed that less than 30% of farmers received specific training on pesticides, with lack of knowledge and use of protective equipment, suggesting that farmers are not properly served by specialists [59]. In the last decade, data released by the Centro Nacional de Toxicología (National Toxicology Center) reported an increase in the number of pesticide poisonings, mostly pyrethroids, organophosphates, carbamates, and organochlorines [66].

Similarly, it is unknown if the early detection programs have prevented a pest from establishing. Despite the fact that the organizational structure and pest identification process has been described, little is known on how this operates at Points of Entry, including identification capacity of potential pests, inspections, fumigation, as well as response times. Pest identifiers at Points of Entry and the LAPROSAVs lack essential resources such as entomological collections. Reference collections should be the core of the national capacity for pest identification and management, but they are not developed enough to support that role in Cuba [67]. Moreover, Cuba has trade with Europe, and the latter follows strict guidelines related to phytosanitary inspection and control [68]. However, it is unclear if novel pests can be identified by the system, and what early detection and rapid response system is in place.

The experience accumulated from agroecological initiatives in thousands of small-and-medium scale farms are critical in the definition of Cuban national policies to support sustainable agriculture. However, in what is known as the ‘Cuban agriculture paradox’, Cuba still imports substantial amounts of food and uses, posing the question of the efficiency of the sustainable programs [55,69]. Policy makers seem to support conventional agriculture when the financial situation improves, while sustainable approaches and agroecology are considered alternatives, only supported under scenarios of economic scarcity.

### Federal and State plant health expert’s perspective: Identifying synergies

Participants from the workshop held at the University of Florida during March 2018 had little previous understanding of the inner workings of the Cuban plant health system. Members from U.S. Federal and State of Florida plant health agencies that participated in the workshop, stressed the need to create partnerships, build trust, and support joint efforts to obtain a clearer sense of how their system operates. Despite the concerns regarding identification capacity, generational gaps, and capacity to respond to a new threat, the strength of the SEPP and pest surveillance was recognized by the participants. Politics or the political climate was identified as a significant barrier for free information sharing between both systems. Science and politics are intimately related given the reciprocal effects that one can have on the other, adding further motivation to build trust between the Cuban and United States phytosanitary systems.

The strength of Cuba’s institutional structure (i.e., the presence of strong organizational structure and the broad geographical coverage) was mentioned as positive and surprising information by workshop participants. The fact that Cuba conducts a non-regulatory National Pest Survey for producers was also highlighted.

Participants identified several knowns and unknowns of Cuba’s phytosanitary system. The structure of the system, which was presented in detail to participants, was identified by all participants as clear. Participants also highlighted that the phytosanitary system presents stable institutions and has a good amount of manpower working on the field. However, there are multiple unknowns about how effectively the system’s structure actually operates. The Cuban system relies on a train-the-trainer type of approach and it is unclear how effective that approach is. In addition, several questions exist regarding how the different ports operate. For example, it was mentioned that the number of tree pests detected over time in Cuba seems too low (based on experiences from other similar settings).

The number of people on the ground and their willingness to engage and collaborate was recognized as a strength of the Cuban system, with individuals immersed in a robust network. When dealing with new quarantine pests, local control measures can be applied if needed. However, people lack diagnostic tools and equipment, and updating their identification capacity was identified as a priority, as they lack access to technologies to effectively share and receive information. Even though the current process works with what they have, dealing with new pest introductions can be slow and inflexible with a decision-making process too far from the ground.

The large number of Cuban ports and airports (~30) in relation to the country’s size, represents a major concern of plant health vulnerability. Little is known about how inspections are conducted at Points of Entry and if current identification capacity can early detect new interceptions or establishments. The Cuban system has a considerable number of individuals working on pest identification and treatment, but it seems to lack the pest control technology required by effective early warning and forecasting systems. The generational gap in knowledge of Cuban scientists was identified both as a potential risk and an area of collaboration, “they are very well trained in pests that have been around for decades, but not so on novel and emerging pests. Many of the lead Cuban scientists were trained in Eastern Europe during the 1990s, and the new generations of scientists need to be trained on these topics.” The scarce pest control technologies and resources, together with the lack proper training, creates lower confidence on Cuban pest lists, perceived by participants as less reliable when compared to other countries.

In the same context, a participatory workshop was held in Cuba to address phytosanitary collaboration opportunities between the two countries during 2018, with participants from the USDA, INISAV, and the University of Florida. A variety of strengths and areas of opportunity were identified for both systems. The perceived strengths of the United States system include flow of scientific information across all levels for decision-making processes, a robust infrastructure and access to state-of-the-art technology, highly trained human resources, and collaboration with national and international entities, while the strengths for the Cuban system mainly concentrate on trained human capital, collaboration between national institutions, and the surveillance system. The main strength of the Cuban system regarding collaboration resides in the strong exchange of information among research centers, done with participatory approaches. However, workshop participants suggested that the system should enhance the relationship between research and industry, as well as its international collaborations. The commercial restrictions imposed by the embargo were identified as the main barrier for scientific and commercial exchange between Cuba and the United States. Similarly, the process followed by the United States phytosanitary system to allow the import of agricultural products is perceived as lengthy, challenging, and highly bureaucratic.

### Comparing NPPOs in the United States and Cuba systems

The main actors of the plant health systems in Cuba and the United States are shown in Table 1. The plant health system in Cuba is under the responsibility of the MINAG, with the DSV as the NPPO. The SEPP is composed not only of the DSV, but also the agrarian sector, the INISAV and other research institutes, and several actors in the Ministry of Education, among other Ministries. In the US, the two agencies in charge of plant biosecurity are the Customs and Border Protection (CBP) agency of the United States Department of Homeland Security (DHS) and the Animal and Plant Health Inspection Service’s Plant Protection and Quarantine (APHIS-PPQ) of the USDA, with USDA-APHIS as the NPPO.

**Table 1:** Organizational structure and decision making in the plant health systems of Cuba and the United States.

Function	Cuba	United States
Plant Health System governance	Ministry of Agriculture (MINAG)	United States Department of Agriculture (USDA)
National Plant Protection Organization (NPPO)	Directorate for Plant Health (DSV)	USDA Animal and Plant Health Inspection Service
Quarantine	Plant Quarantine Central Laboratory (LCCV)	Customs and Border Protection (CBP)
Pest identification	Plant Quarantine Central Laboratory (LCCV)	USDA Animal and Plant Health Inspection Service
	Plant Health Provincial Laboratory (LAPROSAV)	State Department of Agriculture
	Plant Protection Territorial Stations (ETPP)	
Scientific and technical support	Plant Health Research Institute (INISAV)	USDA Center for Plant Health Science and Technology (CPHST)
		USDA Agricultural Research Service (ARS)
		Universities
Extension	Plant Protection Territorial Stations (ETPP)	County Extension Units
	Plant Health Provincial Laboratory (LAPROSAV)	
Training	Plant Health Provincial Laboratory (LAPROSAV)	Land-grant Universities
	Plant Protection Territorial Stations (ETPP)	
	Agrarian Polytechnic Institutes	

Despite NPPOs in both Cuba and the United States focus in biosecurity, different programs and frameworks have been developed. In the United States, the biosecurity (or safeguarding) continuum, a framework has been developed to reduce negative impacts of invasive species [70]. This framework does not only focuses in border inspections, quarantine, eradication, and management, but also include offshore activities. Offshore activities are based on building international capacity, preclearance, and developing international phytosanitary standards. The APHIS International Services program conducts trainings and technology transfer to developing countries to build their plant health infrastructures, reducing the risk that pests will reach the United States.

As a first line of defense against invasive pests in the United States, inspections are conducted by the CBP at seaports, airports, and land border crossings, targeting high-risk hosts and commodities. The PPQ division is responsible for avoiding the entry and establishment of exotic pests, analogous to the LCCV in Cuba. The Cooperative Agricultural Pest Survey (CAPS) from APHIS, which conducts national and state surveys, together with the Forest Service’s Early Detection and Rapid Response program (EDRR), are responsible for post-introduction detection of pests [71], analogous to the surveillance tasks of the ETPPs and LAPROSAVs. Decision making for quarantine purposes in the plant health system of the United States is based on a top-down system, whereas in Cuba, it is a bottom-up system, with a network of institutions that discuss and approve decisions simultaneously. In Cuba, the information moves from the municipal to provincial, to the national level, whereas in the United States, state offices alone can take quarantine decisions to mitigate pest introductions.

The LAPROSAVs and ETPPs, have, in addition, similar extension tasks analogous to the United States county extension offices. The INISAV, responsible for research and development of technologies in Cuba, has similar roles to the USDA Center for Plant Health Science and Technology (CPHST), which provides scientific support for PPQ regulatory decisions and operations, and the USDA Agricultural Research Service (ARS), which conducts research to develop and transfer solutions to agricultural problems (Table 1).

## Discussion

Industrialized agriculture, adopted for example by the United States and many other countries, is characterized by large-scale monocultures and heavy use of synthetic pesticides and chemical fertilizers. Cuba's food crisis led to a policy shift to increase agricultural production without using fossil fuels and heavy machinery. Cuban agroecological production and the innovative farmer organizational schemes, with thousands of farms distributed among cooperatives and individuals after the agrarian decentralization policies, have been of interest in the region, as no other country has achieved these levels of food production with low input agriculture. Moreover, Cuba is internationally recognized as a leader in climate-resilient agroecology and is not a significant contributor of greenhouse gas emissions [72].

Adequate phytosanitary surveillance, which relies on sanitary and phytosanitary capacity, is key to successful national plant protection and international trade. Cuba has a solid organizational structure and legislation to support its Plant Health System. After the Revolution of 1959 and the subsequent establishment of a new agrarian policy, Cuba developed a mature and widespread network of research institutes, laboratories, stations, and human capital to assist plant health issues. The SEPP integrates different research institutes from different Ministries with a coordinated approach. The presence of these institutions, staffed by professionals and technicians from different plant health areas, defined hierarchies, and established roles and responsibilities, reinforces the preventive role of the system. However, key problems were identified across the SEPP that have a significant effects on how the system operates: Obsolete equipment, lack of digitalization, and financial constraints, impacts communication between laboratories and other institutions. These problems delay the response process when rapid response to face new potential threats is required.

A key aspect of the Cuban phytosanitary system is its pest and disease monitoring system that covers most economically important crops in the country, providing pest thresholds to farmers. Human capital is one of the main and most significant assets in the SEPP, with high numbers of professionals, technicians, phytosanitary activists, and farmers, who receive continuous training through dissemination campaigns. Nevertheless, training programs do not always reach all farmers and proper training is lacking in relevant control strategies. Improved training to farmers on pesticide safety and other management methods should be considered to prevent adverse health effects and reduce ecological impacts.

Equally important is the plant quarantine system at Points of Entry, where inspectors follow standardized procedures and are extensively trained. However, there is high uncertainty about how inspections are conducted at Points of Entry, given the lack of essential resources for conducting proper identification. Moreover, identification capacity for current relevant invasive pests in the region was not robust in Cuba, suggesting that new threats could potentially established while being unnoticed.

Preventing the arrival of invasive species through a proactive approach is the most cost-effective way to reduce its impacts. Different options have been implemented by countries to prevent the arrival of a species, including restrictions on trade in species of concern, requirements on exporters to ensure that invaders do not leave their shores or do not survive transit, and the operations of quarantine and inspection facilities at the point of entry [73]. The IPPC standards provide the path for countries to comply with international regulations to manage pest risk and threats to plant health [15]. However, it is up to each country to implement their own satisfactory policies and mobilize sufficient resources to comply with these obligations according to their local needs and capacities. The phytosanitary system in Cuba follows IPPC requirements for phytosanitary certification [74], as has been confirmed by contacted plant health experts, which includes a NPPO with legal, administrative, and operational resources, and infrastructure capabilities [75]. A noticeable sign of this strength is evidenced by the agricultural exports to several IPPC contracting countries across Europe, Asia, and Latin America [76], with Cuban agricultural products that meet phytosanitary requirements demanded by the international market.

Cuban agriculture has been shaped tremendously by larger economic and political forces, with crisis and isolation as the main drivers. If the economic embargo established by the United States is lifted, maintaining the current agricultural development model will be a challenge. With the objective of guaranteeing food security, there is still interest in prioritizing food production, with high external input systems, monoculture methods, and synthetic chemicals. New challenges associated with an expanding economy may push Cuba back into an industrialized agricultural model, and new harms to human health and the environment may arise that are very difficult to manage. Despite natural disasters and instability of international markets, data suggest that Cuba’s food import dependency has been falling for decades [55], with similar food import values to those of many developed countries, rather than developing countries [72].

### Next steps and further collaboration

Several suggested next steps toward future US-Cuba cooperative efforts on plant health emerged from the information collected during workshops, the SWOT analysis, and other sources described herein:

- Identify pathways to build the trust required for effective cooperation in the United States-Cuba relationship, including access to networks and data.
- Further implement suggestions already highlighted in the USDA-MINAG memoranda of understanding and joint statement from 2015 and 2016.
- Identify different channels to facilitate cooperation, especially with regard to travel documents and authorizations.
- Select one hypothetical trade crop from Cuba as an example, such as mango, to build a case study that tracks the steps for permitting/export to the United States. Have people on both sides mapping the process and identifying what would have to happen for this hypothetical case to be viable.
- Focus on activities at the Ports of Entry to understand how inspection and quarantine work.
- Further investigate domestic pest and disease surveillance, from how it is done to how eradication works.
- Listen to, and discuss, the needs and goals of the counterparts in the Cuban system. When Cuba was affected by the screwworm, they requested US help with an eradication program and were turned down. Such actions derail collaboration efforts and are very difficult to overcome.
- Identify if Cuba has harmonized information with other countries (i.e., digital data, not paper) and whether the information can be accessed.
- Establish and fund an academic research post that would liaise with Cuban scientists on agricultural pest and disease solutions to promote information sharing and conduct annual meetings.

Implementation of a proactive regulatory system is highly beneficial, with lower costs of assessing the risks of trade and known impacts. The economic gains associated with improvements to current proactive measures, such as the reinforcement of ISPM 15, would greatly outweigh the cost of early detection programs [77]. However, known impacts and risk are based on reliable information of commercial trade partners.

Understanding structural differences between the Cuban and US systems is highly important as we need to define what is possible in terms of future cooperation. Increasing knowledge of what was once considered a “black box” involves not only engagement, but it is also time consuming and is based on creating one to one relationship between scientists of Cuba and the United States. This is highly beneficial for the United States, as it is a prerequisite for reduction of pest pressure from a very close neighbor, particularly for Florida and other southeastern States [17]. A strong system of pest monitoring and response in Cuba, coupled with sharing of information between the United States and Cuba is critical to preventing new pest invasions between the Caribbean and Florida.

Cuba is a large country in the Caribbean region with a Plant Health System that has evolved over the last 100 years, and, despite its economic struggles, has a strong plant health system that could nevertheless benefit from regional collaborative agreements. The experience and knowledge accumulated in Cuba can serve, in part, as a model for other plant health systems in the region seeking to create a cost-effective system with sustainable benefits. Enhanced communication and collaboration between researchers, agencies, and organizations is key to designing effective solutions against biological invasions [78]. The information retrieved by the workshops showed that there is a high level of uncertainty and even mystery regarding the inner workings of the Cuban phytosanitary system that disrupt effective collaboration. This lack of transparency is understandable considering the key role that this system plays in food security and, therefore, national security. However, the stakes of not engaging in collaboration are very high, and so nascent US-Cuba scientific collaborations must be strengthened through a trust-building process involving established and reliable networks focused on phytosanitary science not politics.

## Conclusions

Phytosanitary systems are extremely important for controlling invasive species risk and plant health to protect agricultural and environmental systems. Prior to this project, very little was publically known about the Cuban phytosanitary system, leading it to be considered as a ‘black box’ by several countries in the region. Through this study, scientists in the US and Cuba developed shared understandings of their respective phytosanitary systems–information that is critical for safeguarding against invasive species, and also for potentially normalizing trade between the two countries. We provided a descriptive analysis of the characteristics and potential impacts of their organizational structure and diagnostic capacity on the regional economy, identifying important steps for further cooperation. Despite several shortcomings, the inherent strengths in the Cuban plant health system should benefit the region. However, some aspects of the Cuban phytosanitary system constrain its full potential. For example, key problems such as obsolete equipment and lack of digitalization were found. Moreover, it is unclear if pest management and early detection programs meet their goals, with repercussions to plant protection issues in the Caribbean region.

The impacts of invasive species are typically not incorporated into the costs of trade. These costs are spread widely across society as government agencies spend on inspection, interception, eradication, containment, and compensation. Reducing the impacts of these species will require agreements that allow for internationally coordinated action. Next steps should focus on improving collaboration, building trust, and focusing on capacity building, as understanding the effectiveness of phytosanitary system is crucial for regional economic stability of a post embargo United States-Cuban relationship.
